# Maculopathie chez une patiente lupique après la prise des antipaludéens de synthèse

**DOI:** 10.11604/pamj.2014.18.146.4026

**Published:** 2014-06-17

**Authors:** Ryme Abdelkhalek, Yousra Hanafi

**Affiliations:** 1 Service d'Ophtalmologie, Hôpital Militaire d'Instruction Mohamed V, Rabat, Maroc

**Keywords:** Maculopathie, lupus, antipaludéens de synthèse, Maculopathy, lupus, antimalarial drugs

## Image en medicine

Il s'agit du cas d'une patiente âgée de 52 ans, aux antécédents de lupus érythémateux systémique(LES) évoluant depuis 1997, actuellement sous Nivaquine (APS) à la dose de 2 comprimés par jour, adressé pour baisse de la vision bilatérale. L'examen de l'oeil droit trouve une vision corrigée à 1/10, un segment antérieur d'aspect normal et au fond d'oeil une maculopathie en cocarde (A). L'examen de l'oeil gauche trouve une vision corrigée à 2/10 faible, un segment antérieur calme et au fond une maculopathie en cocarde. L'angiographie rétinienne à la fluorescéine montre une image en oeil-de-boeuf bilatéral (B).

**Figure 1:**
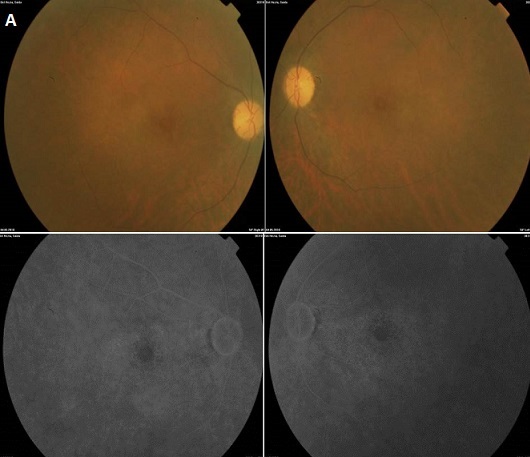
A) fond de l’œil montrant une maculopathie en cocarde bilatérale; B) L'angiographie à la fluorescéine montre une image en œil-de-bœuf bilateral

